# Wellbeing at Work before and during the SARS-COV-2 Pandemic: A Brazilian Nationwide Study among Dietitians

**DOI:** 10.3390/ijerph17155541

**Published:** 2020-07-31

**Authors:** Raquel Adjafre da Costa Matos, Rita de Cássia Coelho de Almeida Akutsu, Renata Puppin Zandonadi, Ada Rocha, Raquel Braz Assunção Botelho

**Affiliations:** 1 Department of Nutrition, Faculty of Health Sciences, University of Brasilia, Brasilia 70910-900, Brazil; raquel.adjafre@gmail.com (R.A.d.C.M.); rita.akutsu@gmail.com (R.d.C.C.d.A.A.); renatapz@yahoo.com.br (R.P.Z.); 2 Faculdade de Ciencias da Nutrição e Alimentação, University of Porto, 4200-464 Porto, Portugal; adarocha@fcna.up.pt

**Keywords:** SARS-COV-2, dietitians, pandemic, wellbeing at work

## Abstract

This study aimed to evaluate the perceptions of dietitians’ wellbeing at work before and during the SARS-COV-2 pandemic in Brazil. This cross-sectional study was performed using a previously validated instrument to investigate the wellbeing of dietitians at work in Brazil. The questionnaire on the wellbeing of dietitians was composed of 25 items (with a 5-point scale), characteristics, and questions about the SARS-COV-2 period. The application was carried out with GoogleForms^®^ tool from 26 May to 7 June 2020. The weblink to access the research was sent via email, messaging apps, and social networks. Volunteers were recruited nationwide with the help of the Brazilian Dietitians Councils, support groups, as well as media outreach to reach as many dietitians as possible. Volunteers received, along with the research link, the invitation to participate, as well as the consent form. A representative sample of 1359 dietitians from all the Brazilian regions answered the questionnaire—mostly female (92.5%), Catholic (52.9%), from 25 to 39 years old (58.4%), with a partner (63.8%), and with no children (58%). Most of the participants continue working during the pandemic period (83.8%), but they did not have SARS-COV-2 (96%), nor did their family members (80.7%). The wellbeing at work before SARS-COV-2 was 3.88 ± 0.71, statistically different (*p* < 0.05) from during the pandemic, with the wellbeing of 3.71 ± 0.78. Wellbeing at work was higher before the pandemic for all the analyzed variables. Analyzing variables separately before and during the pandemic, dietitians with partners, children and a Ph.D. presented higher scores for wellbeing at work. Professionals receiving more than five times the minimum wage have higher scores. During the pandemic, better wellbeing was observed for dietitians working remotely.

## 1. Introduction

The world is facing the unexpected SARS-COV-2 pandemic with several consequences for economic, social, mental, environmental, and health aspects. The SARS-COV-2 brought not only the risk of death from the viral infection but also unbearable psychological pressure on people in the world [1]. According to the World of Health Organization (WHO), until June 7, 2020, 6,799,713 cases of SARS-COV-2 were registered and there have been 397,388 deaths in the world [2]. Americas represent almost half of the registered cases (*n* = 3,234,875 cases; 47.6%) and deaths (*n* = 179,394 deaths; 45%) in the period. These numbers could be underestimated due to the lack of medical diagnosis at the beginning of the pandemic. Some countries face a few diagnostic tests. In South America, Brazil is the country with the most registered cases of SARS-COV-2 (60.3%, *n* = 645,771, until 7 June 2020) and deaths (72.7%, *n* = 35,026 in the same period) [3].

In Brazil, on the frontline of coping with SARS-COV-2, health professionals are acting on different fronts. To date, the future has been uncertain, and healthcare professionals have stepped out of their comfort zones [4]. There are several scenarios faced by health care professionals, especially ones working in clinics or hospitals, with the risk of catching SARS-COV-2, and the ones that are experiencing unemployment and family income reduction without knowing how they will face the economic crisis during and after the pandemic. In this sense, during the pandemic period, they may experience maladaptive psychological consequences of their jobs [5]. Work plays a central role in the individuals’ lives, bringing paradoxical consequences to the social, physical, and psychological integrity of workers [6,7], and low job satisfaction is the leading cause of turnover among health care professionals [8,9]. During the pandemic period, this can get worse.

As well as some other healthcare professionals, during the pandemic, dietitians may be facing some difficulties in their jobs, beyond the traditional ones such as low payment, lack of professional recognition, difficulty in getting their first jobs, and difficulty in geographical mobility [10,11,12,13]. In Brazil, dietitians present a wide range of work options, from directly working in hospitals visiting patients or conducting hospital foodservices, to working in clinics, schools, and commercial restaurants. All of these environments can bring direct contact with possibly infected people, presenting challenges in this new work scenario. The number of patients in the hospitals increased, and the way the food is served needed to change. Restaurants outside the hospitals had to close their doors, leading to unemployment, and facing many risks in reopening when permitted. It is essential to highlight those dietitians, despite being a specific niche of the health system, that are working directly with people infected with Sars-CoV-2. Often, their risks are ignored or underestimated by other health professionals and even by the Federal Government, which can negatively impact wellbeing at work. Recent legislation published on July 8, 2020 (Law No. 14.023) [14] dealing with the protection of professionals exposed to the risks of Sars-Cov-2, did not include dietitians on its lists.

Despite the enormous gathering of scientific data, to date, there is no treatment or vaccine for SARS-COV-2. This scenario of uncertainties about the disease, security itself, and their jobs can affect the perceptions of dietitians about their wellbeing at work. Studies conceive wellbeing at work as a process, defining it as the satisfaction of needs and the fulfillment of individuals’ desires as they fulfill their role in the profession [15,16]. This conception considers the role of work organizations in the health of individuals, and the development of healthy environments enables positive relationships and attitudes. The wellbeing of a professional category can be impacted, among other things, by the general and work values that are expressed in professional practice and social coexistence [16]. Both of them can be facing negative impacts during the SARS-COV-2 pandemic. Wellbeing at work has a significant impact on work performance and quality of life, and it brings paradoxical consequences for the social, physical, and psychological integrity of workers [6,7]. Low wellbeing at work and low job satisfaction are considered the leading causes of turnover among health care professionals [8,9]. Therefore, the knowledge about wellbeing at work is vital to improve the working environment and the quality of the service [8]. Wellbeing at work is related to lower levels of stress, work-related diseases, burnout, depression, and unhealthy personal practices (smoking, drinking, overeating, lack of exercise), and consequently, lower levels of non-communicable chronic diseases (NCD) [17]. There have been studies on the psychological impact of the epidemic on the general public, patients, medical staff, children, and older adults [1,18,19]. However, there is no study on the perception of dietitians’ wellbeing at work during the difficult times of pandemic. The hypothesis is that wellbeing at work will decrease during the pandemic period. In this sense, this study aimed to evaluate dietitians’ perceptions of wellbeing at work before and during the SARS-COV-2 pandemic in Brazil. The professional wellbeing knowledge among dietitians may lead to effective avenues to prevent or manage stress, unhealthy personal practices, and NCD. By evaluating the period before and during the SARS-COV-2 pandemic period, we expect that a clear understanding of the factors that influence dietitians’ wellbeing in these two moments may contribute to helping these professionals to recover after the pandemic period, exploring the areas that were most affected, and working on better professional valorization, improving the public’s trust in dietitians and the dynamics of the interprofessional healthcare team.

## 2. Materials and Methods

### 2.1. Study Design and Instrument

This exploratory and cross-sectional study was performed using a previously validated instrument [16] to investigate the wellbeing of dietitians before and during the SARS-COV-2 pandemic in Brazil. The questionnaire was composed of 25 items on the wellbeing of dietitians (with a 5-point scale that varies from 1 to 5). It also included characteristics from the original study [16] such as gender, age, marital status, the Brazilian state of current residency, religion, number of individuals living in the house, family income, children, educational level, occupational area as a dietitian, number of workplaces as a dietitian, how long ago graduation ended, and type of university. Researchers included three questions about the SARS-COV-2 period: Do you continue working during the SARS-COV-2 pandemic? Did you test positive for SARS-COV-2? and Did anybody in your family test positive for SARS-COV-2? The complete questionnaire is available in Appendix A.

The instrument application was carried out with GoogleForms^®^ tool (Google LLC, Mountain View, CA, USA) from May 26 to June 7, 2020. The weblink to access the research was sent via email, messaging apps, and social networks. Volunteers were recruited nationwide with the help of the Brazilian Dietitians Councils, support groups, and media outreach to reach as many dietitians as possible. Volunteers received, along with the research link, the invitation to participate, as well as the consent form.

### 2.2. Participants and Ethics

Dietitians from the entire country were recruited to participate in the study. Researchers wanted to trace the wellbeing at work before and during the SARS-COV-2 of this population group in Brazil. Ethical approval was obtained for this study by the Ethics Committee University of Brasília (protocol No. 54822316.1.00000030). The study was conducted according to the guidelines laid down in the Declaration of Helsinki and followed the Recommendations for the Conduct, Reporting, Editing, and Publication of Scholarly work in Medical Journals.

The sampling size was calculated based on data from the Brazilian Federal Dietitians Council that presents 129,134 registered dietitians [20], considering an error (e) of 3% and a level of significance (α) of 5% [21]. The minimum estimated representative sample size would be of 1059 participants. The inclusion criteria were to be a dietitian and living and working in Brazil.

### 2.3. Statistical Analysis

Researchers extracted data from the GoogleForms^®^ tool and analyzed using Statistical Package for the Social Sciences—SPSS 24.0 (version 24, SPSS Inc., Chicago, IL, USA). Exploratory and confirmatory analyses were conducted to determine the psychometric quality of the wellbeing at work instrument. We used the Kaiser–Meyer–Olkin (KMO), and Barlett’s sphericity test. For consistency, Cronbach’s alpha was used. Descriptive analyses were used to determine the measures of central tendency and dispersion of the sample. We compared means of the sample through paired *t*-tests (wellbeing before and during SARS-COV-2) and Analysis of Variance (ANOVA) with Tukey post-hoc.

## 3. Results

**Figure 1:**
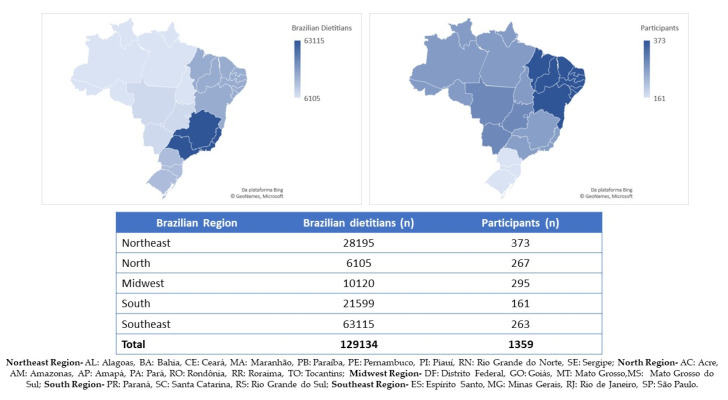
Distribution of dietitians and participants among Brazilian regions.

A representative sample of 1359 dietitians from all the 26 Brazilian states and the Federal District and regions answered the questionnaire. Figure 1 shows the distribution of the dietitians and participants by Brazilian regions.

The participants were mostly female (92.5%), Catholic (52.9%), aged from 25 to 39 years old (58.4%), with a partner (63.8%), and with no children (58%) (Table 1). Most of the participants continued working during the pandemic period (83.8%), but had not been diagnosed with SARS-COV-2 (96%) before answering the questionnaire, nor did their family members (80.7%).

Dietitians with less than 1 MW family income are mostly graduates (35.9%) or present specialization/residency (56.4%). However, dietitians with more than 20 MW as a family income have a master’s and Ph.D. (52.2%). Of 144 dietitians with Ph.D., 95.1% work in the teaching area (universities).

Table 2 shows data from the wellbeing at work before and during the pandemic period compared by participants’ characteristics. The instrument presented a KMO of 0.957 and a significant (0.000) Barlett’s sphericity test. For internal consistency, the wellbeing at work instrument presented a Cronbach’s alpha of 0.952. In the communalities, the extraction item was below 0.500 for questions 12 (social relations with my colleagues positively influence my work) and 25 (I consider my workload adequate), and they were not considered for the final score of wellbeing at work. The maintained questions from the instrument were divided into four factors. Factor 1 is related to exterior perception with questions 2, 4, 6, 7, 11 and 15 (Appendix A), factor 2 is concerned about the perception in itself (questions 1, 3, 5, 8, 13, 14, 18 and 21), factor 3 is the task perception (questions 19, 20, 22, 23 and 24), and factor 4 is the perception from the dietitians’ category (questions 9, 10, 16 and 17). Cronbach´s alpha was also calculated for each factor: factor 1, 0.871; factor 2, 0.881; factor 3, 0.881; factor 4, 0.884 (Supplementary File, Table S1).

In general, wellbeing at work before SARS-COV-2 was 3.88 ± 0.71, statistically different (*p* < 0.05) from during the pandemic, with the wellbeing of 3.71 ± 0.78. A comparison between before the pandemic and during the pandemic showed statistical differences for all variables (worse during the pandemic period) (*p* < 0.05).

Gender and number of workplaces did not influence wellbeing at work before and during the pandemic period. Individuals with a partner and with children had a better perception of wellbeing at work than the ones with no partner or children. Before and during the pandemic, master’s and Ph.D. individuals presented better wellbeing at work than graduates, and Ph.D. dietitians presented better wellbeing than dietitians with a master’s degree (Table 2).

For both periods, individuals that work in teaching present better wellbeing at work compared to the other areas of dietitians’ practice (clinic, foodservice administration, public health, and others). Before the pandemic period, individuals with family monthly income >5 MW present higher wellbeing at work than the ones up to 5 MW (Table 2). During the pandemic period, the results were a little different, showing differences among individuals up to 3 MW, from 3 to 5 MW and >5 MW, with increasing wellbeing perception with higher family income. Before the pandemic, the time from the undergraduate completion differed from up to 10 years to >15 years. During the pandemic, the higher time from undergraduate completion (>15 years) presented a higher mean of wellbeing at work than the lowest time of completion (≤2 years).

Before the pandemic period, individuals adept in Catholicism and Spiritism had a better perception of wellbeing at work than Protestants. During the pandemic, Catholics did not differ from Protestants, agnostics, or Spiritism followers. However, individuals following the Spiritism religion presented a better perception of wellbeing at work than Protestants (Table 2).

Dietitians that tested positive for SARS-COV-2 (*n* = 55) were predominantly working in-person (78.2%, *n* = 43), without or with adaptations (58.2%, *n* = 32; 20%, *n* = 11, respectively). Participants who reported not being working during the pandemic period (*n* = 220) have a job, but they are unable to work in-person or remotely. The wellbeing values were considered for this group of workers because they answered the questions and effectively have a job. There is no difference among perceptions of wellbeing at work between dietitians who had SARS-COV-2 and the ones that did not have, similarly to the results from the ones that had any family member test positive for SARS-COV-2.

When separating the wellbeing at work by factors (Table S1) and comparing by participant´s characteristics, factors 1 and 4 presented the lowest means before and after the pandemic, being lower and statistically different (*p* < 0.05) during the pandemic. Factor 1 relates to exterior perception and factor 4 to the category perception. Higher scores occurred for the perception of itself and the perception of the task, before and during the pandemic.

## 4. Discussion

As the world grapples with the impact of the SARS-CoV-2 pandemic, health care workers face extraordinary challenges daily, in different contexts and conditions [22]. The media reports that health care professionals are hugely concerned for the health and wellbeing of their patients, their families, and themselves, facing pandemic issues [22,23]. However, the Brazilian government did not recognize dietitians as part of the health professionals facing the SARS-COV-2 pandemic [14]. These changes in life and work and the lack of recognition and support have a significant impact on their wellbeing, as shown by our results. Among Brazilian dietitians, there was a worse perception of wellbeing at work during the pandemic compared to the period before the pandemic for all variables (*p* < 0.05) (Table 2). In general, dietitians’ wellbeing at work was positive (above 2.5), which is the midpoint of the scale. The items that obtained the best scores were those that investigate the perception of the importance of the profession for themselves and society. The average scores were above 4.40 before the SARS-COV-2 pandemic and 4.20 during it. The items with the lowest scores and which need to be improved are related to compensation and technological support to perform the tasks assigned to the dietitian. Probably, wage improvement could come through professional qualification, as higher wages were linked to more years of study in our research. Besides, there is a need for an increase in the number of class entities (unions and councils) to fight for better wages of this professional category. In Brazil, even before the SARS-COV-2 pandemic, the country had high unemployment rates [24], a situation worsened by the pandemic, which made it difficult for workers to maintain their family income. Part of the dietitians’ work is in the foodservice area (schools, commercial and institutional restaurants), and most of these are closed or changed the policies of production due to the food safety and workers’ safety conditions.

Wellbeing at work before SARS-COV-2 was 3.88 ± 0.71, statistically different (*p* < 0.05) from during the pandemic (3.71 ± 0.78). Usually, these conditions would place health care professionals in a situation defined by threat and fear, both of which have been shown to have a detrimental effect on their ability to offer compassionate and person-centered care to their patients [22,25]. In these circumstances, there are high levels of conflict within teams and workplace adversity, leading to a working environment which is perceived as hostile, abusive, and unrewarding [26,27]. As a consequence, workplace adversity can be correlated with a decreased quality of care [28]. In April 2020, the Brazilian Health Ministry requested the registration of all health professionals, including dietitians, to reinforce the fight against SARS-CoV-2, in addition to the usual work of individuals [29]. This reinforcement is to assist managers of the Unified Health System (*SUS*) in coping with SARS-CoV-2, based on the work capacity of these professionals. It focuses on those who were available to go to the Brazilian states with the greatest need to strengthen health teams [29]. This fact also caused concern in several dietitians who were afraid to work facing SARS-COV-2 patients for various reasons. According to the Brazilian Health Ministry, from the 90,245 Brazilian registered dietitians to reinforce the fight against SARS-CoV-2, 33,624 were willing to work facing SARS-COV-2 [30]. These professionals were registered in the category to receive payment for their work to confront SARS-COV-2. However, the Brazilian Ministry registered other health care professions in a voluntary (unpaid) category of workers, and no dietitian volunteered until 28 April 2020 [30]. It was not stated, but it seems that the dietitians that were willing to work facing SARS-COV-2 were doing it to help the family income, which can potentially worsen their perception of wellbeing at work. Given the unknown and uncontrollable nature of the SARS-COV-2, some health care professionals need to stay away from their home and loved ones, possibly affecting emotional aspects and the relationship with their work [23].

In the pandemic situation, the protection measures for these professionals get worse due to the limitations of social distance. In some cases, this risk is higher, such as in hospitals, emergency services, outpatient clinics, vaccination clinics, screening lines, and other health care settings. In this environment, these professionals are even more exposed when their duty includes providing some assistance to infected people with SARS-COV-2. In foodservices, where these professionals do not work directly with individuals adequately tested for the new coronavirus, they face the same environment with asymptomatic individuals or those in the incubation phase of the disease [31].

Our sample was mostly composed of females (Table 1). The female hegemony among dietitians is common, as shown by other studies in different countries [8,9,13,32,33,34]. Female hegemony in the profession can represent repercussions on career, social prestige, and income [12]. A study showed that dietitians were mostly women and that, although the labor market has grown, the new jobs were mostly (86%) part-time, not only because women need to conciliate career and family care, but also because these positions get lower payment [35]. However, according to our data, gender did not influence wellbeing at work before nor during the pandemic period.

According to a research conducted by the Dietitians Federal Council in 2016/2017 with 1104 dietitians in Brazil, most of them are young, between 25 and 44 years old (81%), a higher percentage than our study (68.3%). Previous studies showed that 73% of the Brazilian dietitians are postgraduates [12,16,36], similar to our data (77.8%, *n* = 1058). Before and during the pandemic, master’s and Ph.D. individuals presented better wellbeing at work than graduates (Table 2). Most Ph.D. dietitians work in the teaching area, and for both periods, individuals that work in teaching present better wellbeing at work compared to the other areas of dietitians’ practice (clinic, foodservice administration, public health, and others). All schools, including universities, faced changes in how they conducted work. Professors had to search for technological tools and strategies in order to work during the pandemic period, impacting their perception of wellbeing at work, as shown by the lower mean during the pandemic. Undergraduate courses in the health area need practical classes and time inside the hospitals, and it is difficult to return to these activities in-person. The other work areas for dietitians did not present statistically different wellbeing, even for professionals that work in the clinical area and can be inside hospitals.

Before the pandemic period, individuals with family monthly income > 5 MW presented higher wellbeing at work than the ones up to 5 MW (Table 2). During the pandemic period, wellbeing perception increased with higher family income. These data are confirmed by other studies, indicating that lower wages decrease satisfaction at work [13,16,37,38,39]. During the pandemic period, most family members are isolated at home, increasing expenses with bills (water, energy, food, and others). This can influence the difference between the categories of family income compared to the period before the SARS-COV-2. According to the official data [24], unemployment increased in all Brazilian regions with SARS-COV-2, but mainly in the northeast region (from 13.6% in 2019 to 15.6% in 2020), followed by the North (from 10.6% to 11.4%) and the southeast region (from 11.4% to 12.4%), also impacting family income. It is noteworthy that the north and northeast regions were the ones presenting the largest proportional increase in official cases of SARS-COV-2 in Brazil until June 7, 2020 [40].

The Brazilian government published two provisional measures [41,42] during the pandemic changing the work relationship between employers and employees. Employers can reduce employees’ salaries during the SARS-COV-2 crisis, and rules were established for remote work and the suspension of some administrative measures related to safety at work. These legislative measures enable up to a 70% salary reduction and precarious work relationships, reflected by a lower perception of wellbeing at work during the pandemic.

Two characteristics influence wellbeing at work before and during the pandemic: marital status, and children. Dietitians with partners (63.8%) and with children (42%) had higher wellbeing before and during the pandemic. VanderWeele [43] discusses in his article that studies associate marriage with higher life satisfaction and happiness. This association can be related to better mental and physical health and longevity. With time, marriage is associated with a better relationship with others, including work partners, which can influence work wellbeing. VanderWeele [43] also highlights that marriage and family are vital to wellbeing. Despite this study evaluating wellbeing in life, not in the workplace, it could potentially explain higher scores of wellbeing in our study for dietitians with children and partners. Wilcox [44] discusses in his book that marriage is also associated with financial status and education. A meta-analytic study [45] suggested that people who are employed present better life, family, and marital satisfaction. Our study was only conducted with dietitians that have a job, because the instrument is related to wellbeing at work. Therefore, it is not possible to compare this with studies of unemployment. However, they show better wellbeing for people with partners, family, and education, such as our findings for wellbeing at work [46]. Ryff and Heidrich [46] stated that work and education experiences explain differences in the purpose of life. Higher levels of education are associated with happiness and satisfaction and strongly affect income [47]. As already discussed, dietitians with more education and income presented better scores for wellbeing before and during the pandemic.

Regarding religion, worldwide, 15% of people are agnostic [48], higher than in our study (5.2%), but closer to the Brazilian statistics of 8% (IBGE, 2010). Even though research has shown that religion brings higher subjective wellbeing because of social support and meaning in life, wellbeing at work was not lower among agnostic dietitians [49]. Sedikides [50] stated that religion is important for most people´s psychological conditions and subjective wellbeing. There are pieces of evidence that suggest that spirituality is a protective factor for health and psychological problems [51,52]. A study conducted by Ferreira, Pinto e Neto [52] with university students from Portugal, Mozambique, Angola, and Brazil showed that churchgoers present better spiritual wellbeing and better life satisfaction. During the pandemic, churches and temples were closed in many cities and states, and this can explain the lower wellbeing in our study for all the religions during the pandemic.

Dietitians that tested positive for SARS-COV-2 (*n* = 55) were predominantly working in-person (78.2%, *n* = 43), without or with adaptations (58.2%, *n* = 32; 20%, *n* = 11, respectively). Despite the need for adaptations, people working remotely during the pandemic had better wellbeing at work than the ones that are working in person. Probably, this is due to the possibility of a greater sense of security at home, and being near the family.

The new coronavirus pandemic arrived in Brazil at a time of economic stagnation, problems with the health and social protection systems, difficulties among the food security programs, accelerated increase in poverty, and, especially, extreme poverty, and a significant increase in the homeless population. Since March 2020, Brazil has accumulated a fall in gross domestic product (GDP) [53], and this retreat, partially caused by social isolation, has significantly increased formal and informal unemployment, in addition to precarious labor relations. This new scenario will directly impact dietitian’s work and wellbeing, not only their work conditions, incomes, and uncertainties, but also the feeling of helplessness when facing hunger in the country.

At the same time, the pandemic can bring a search for new strategies for better conditions for health professionals in hospitals and clinics. New routines and behaviors for food production can be developed, not thinking only of food safety inside the production area, but also the attitudes of consumers. Dietitians have the potential to show the importance of their work, to avoid contaminations in foodservices, and bring more discussion about eating habits and immunity.

Dietitians are health professionals at the front line for the population’s nutritional assistance. They work at all levels of complexity in the health care system, and may potentially reduce the risks of disease worsening, and contribute to the recovery of patients affected by Sars-Cov-2. The different spheres (population, governments, and other health professionals) must recognize the relevance of these professionals for public health in the country.

## 5. Conclusions

These data are essential to evaluating dietitians’ perceptions of wellbeing at work and potentially helping to understand the main challenges supporting them to emerge from the pandemic as a different type of health care practitioner. The instrument presented an excellent KMO and internal consistency as a whole or by its factors. For all the participants’ characteristics, wellbeing decreased during the pandemic, not showing specific influence among the analyzed variables. The hypothesis that the SARS-COV-2 pandemic period influences the wellbeing of Brazilian dietitians was confirmed. However, when evaluating wellbeing separately, before and during the pandemic, dietitians with partners, children, Ph.D., and receiving more than five MW presented higher wellbeing scores at work. During the pandemic period, dietitians working remotely also showed higher wellbeing. Regardless of the period, it is notable that, for dietitians’ wellbeing at work to improve, better compensation for and recognition of the profession is necessary, as well as the conditions for their activities to be carried out. Health policymakers should discuss the role of health professionals as a multidisciplinary team, highlighting the importance of each category for the health system. Much improvement has happened in the health system in Brazil to integrate professionals but, as discussed, dietitians still feel unrecognized for their work. This study can open doors for more research and discussion in the field of wellbeing at work for health professionals, as well as a clear understanding of the factors that influence dietitians’ wellbeing before and during the SARS-COV-2 pandemic, helping these professionals to recover after this period. Therefore, the data could help to explore the areas that most affected (before and during the pandemic period) wellbeing at work, favoring professional valorization. Further studies should be conducted after the pandemic period to evaluate the perceptions of dietitians’ wellbeing at work due to the potential changes in the work environment and conditions in Brazil, and also in other countries, allowing comparisons netweem them.

## Figures and Tables

**Table 1:** Characteristics of Brazilian dietitians and SARS-COV-2 questions (*n* = 1359).

Variable		*n*	%
Gender	Female	1258	92.5
	Male	102	7.5
Age group	21 to 24 y/o	149	11.0
	25 to 29 y/o	275	20.2
	30 to 34 y/o	272	20.0
	35 to 39 y/o	248	18.2
	40 to 44 y/o	134	9.9
	45 to 49 y/o	188	6.5
	50 to older	194	14.3
Religion	Catholic	720	52.9
	Protestant	283	20.8
	Spiritism	213	15.7
	Agnostic	71	5.2
	Others	73	5.4
Level of education (highest degree)	Graduate	302	22.2
	Especialization/Residency	677	49.8
	Master	237	17.4
	PhD	144	10.6
Marital status	Without partner	493	36.3
	With partner	867	63.8
Children	Yes	571	42.0
	No	789	58.0
Family monthly income	≤1 MW	39	2.9
	>1 to 2 MW	111	8.2
	>2 to 3 MW	199	14.6
	>3 tp 5 MW	297	21.8
	>5 to 10 MW	414	30.4
	>10 to 20 MW	229	16.8
	>20 MW	71	5.2
Number of household members	1	140	10.6
	2	366	27.7
	3	370	28.1
	4	320	24.3
	>5	123	9.3
Area of Practice	Clinic	327	24.0
	Foodservice administration	173	12.7
	Public health	117	8.6
	Teaching	99	7.3
	Others	72	5.3
	More than one area of practice	*572*	*42.1*
Number of workplaces	1	858	63.1
	2	353	26.0
	3	85	6.3
	>3	64	4.7
Type of institution where you finished your undergraduate degree	Public	656	48.2
	Private	704	51.8
Time from the undergraduate completion	≤2 years	288	21.2
	>2 to 5 years	232	17.1
	>5 to 10 years	255	18.8
	>10 to 15 years	253	18.6
	>15 years	332	24.4
Do you continue working during SARS-COV-2?	no	220	16.2
	yes remotely	486	35.7
	yes in person with some adaptations	360	26.5
	yes in person	294	21.6
Did you test positive for SARS-COV-2?	No	1304	96.0
	Yes	55	4.0
Did any family members test positive for SARS-COV-2?	No	1097	80.7
	Yes (does not live with me)	69	5.1
	Yes (living with me)	194	14.3

MW—Minimum Wage in Brazil (June 7th 2020)—U$ 213.0.; y/o—years old.

**Table 2:** Wellbeing at work by socioeconomic and demographic variables of Brazilian dietitians before and during the pandemic period (*n* = 1359).

Variable		Before * Pandemic	During * Pandemic
		Mean ± SD	Mean ± SD
Gender	Female	3.88 ^a^ ± 0.71	3.70 ^a^ ± 0.78
	Male	3.92 ^a^ ± 0.71	3.79 ^a^ ± 0.79
Age group	21 to 24 y/o	3.77 ^abc^ ± 0.77	3.63 ^a^ ± 0.82
	25 to 29 y/o	3.79 ^abc^ ± 0.76	3.65 ^a^ ± 0.80
	30 to 34 y/o	3.81 ^b^ ± 0.71	3.64 ^a^ ± 0.75
	35 to 39 y/o	3.94 ^abc^ ± 0.65	3.77 ^ab^ ± 0.78
	40 to 44 y/o	3.90 ^abc^ ± 0.70	3.71 ^ab^ ± 0.81
	45 to 49 y/o	3.98 ^abc^ ± 0.63	3.75 ^ab^ ± 0.81
	50 to older	4.05 ^ac^ ± 0.65	3.86 ^b^ ± 0.73
Brazilian region	North	3.76 ^a^ ± 0.79	3.61 ^a^ ± 0.83
	Northeast	3.86 ^ab^ ± 0.70	3.67 ^ab^ ± 0.77
	Midwest	3.89 ^ab^ ± 0.66	3.69 ^ab^ ± 0.76
	Southeast	3.95 ^b^ ± 0.73	3.81 ^b^ ± 0.80
	South	3.98 ^b^ ± 0.64	3.80 ^ab^ ± 0.76
Religion	Catholic	3.91 ^a^ ± 0.69	3.73 ^abc^ ± 0.77
	Protestant	3.72 ^b^ ± 0.77	3.59 ^b^ ± 0.82
	Spiritism	3.99 ^a^ ± 0.64	3.83 ^c^ ± 0.74
	Agnostic	3.89 ^ab^ ± 0.70	3.71 ^abc^ ± 0.80
	Others	3.88 ^ab^ ± 0.78	3.68 ^abc^ ± 0.88
Level of education (highest degree)	Graduate	3.75 ^a^ ± 0.75	3.57 ^a^ ± 0.80
	Specialization/Residency	3.85 ^ab^ ± 0.71	3.68 ^ab^ ± 0.79
	Master’s	3.97 ^b^ ± 0.68	3.78 ^b^ ± 0.77
	PhD	4.17 ^c^ ± 0.55	4.01 ^c^ ± 0.65
Marital status	Without partner	3.79 ^a^ ± 0.74	3.60 ^a^ ± 0.81
	With partner	3.93 ^b^ ± 0.69	3.77 ^b^ ± 0.76
Children	Yes	3.94 ^a^ ± 0.68	3.77 ^a^ ± 0.75
	No	3.84 ^b^ ± 0.72	3.66 ^b^ ± 0.80
Family monthly income	≤1 MW	3.49 ^a^ ± 0.85	3.29 ^a^ ± 0.90
	>1 to 2 MW	3.53 ^a^ ± 0.80	3.33 ^a^ ± 0.83
	>2 to 3 MW	3.74 ^a^ ± 0.79	3.55 ^a^ ± 0.84
	>3 to 5 MW	3.81 ^a^ ± 0.67	3.61 ^b^ ± 0.78
	>5 to 10 MW	4.01 ^b^ ± 0.63	3.86 ^c^ ± 0.71
	>10 to 20 MW	4.06 ^b^ ± 0.61	3.91 ^c^ ± 0.70
	>20 MW	4.01 ^b^ ± 0.69	3.85 ^c^ ± 0.78
Area of Practice	Clinic	3.87 ^a^ ± 0.73	3.72 ^ac^ ± 0.78
	Teaching	4.20 ^b^ ± 0.55	3.99 ^b^ ± 0.69
	Foodservice administration	3.73 ^a^ ± 0.76	3.58 ^ac^ ± 0.83
	Public health	3.75 ^a^ ± 0.67	3.49 ^ac^ ± 0.77
	More than one area of practice	3.90 ^a^ ± 0.71	3.74 ^ab^ ± 0.78
	Others	3.90 ^a^ ± 0.62	3.69 ^ac^ ± 0.75
Number of workplaces	1	3.85 ^a^ ± 0.72	3.67 ^a^ ± 0.79
	2	3.90 ^a^ ± 0.68	3.74 ^a^ ± 0.75
	3	3.99 ^a^ ± 0.74	3.82 ^a^ ± 0.78
	>3	4.02 ^a^ ± 0.67	3.82 ^a^ ± 0.82
Type of institution where you finished your undergraduate degree	Public	3.92 ^a^ ± 0.67	3.74 ^a^ ± 0.73
	Private	3.84 ^b^ ± 0.74	3.68 ^a^ ± 0.83
Time from the undergraduate completion	≤2 years	3.76 ^a^ ± 0.74	3.59 ^a^ ± 0.83
	>2 to 5 years	3.80 ^a^ ± 0.77	3.66 ^ab^ ± 0.79
	>5 to 10 years	3.85 ^a^ ± 0.72	3.68 ^ab^ ± 0.79
	>10 to 15 years	3.93 ^ab^ ± 0.70	3.76 ^ab^ ± 0.80
	>15 years	4.03 ^b^ ± 0.60	3.83 ^b^ ± 0.71
Do you continue working during SARS-COV-2?	No	3.65 *8 ± 0.76	3.33 ^a^ ± 0.85
	Yes, in person	3.80 *8 ± 0.68	3.65 ^b^ ± 0.72
	Yes, in person with some adaptations	3.90 *8 ± 0.72	3.74 ^b^ ± 0.79
	yes remotely	4.02 *8 ± 0.66	3.89 ^c^ ± 0.72
Did you test positive for SARS-COV-2?	No	3.89 * ± 0.71	3.71 ^a^ ± 0.79
	Yes	3.76 * ± 0.71	3.65 ^a^ ± 0.70
Did any family members test positive for SARS-COV-2?	No	3.89 * ± 0.70	3.71 ^a^ ± 0.78
	Yes (does not live with me)	3.88 * ± 0.64	3.74 ^a^ ± 0.67
	Yes (living with me)	3.84 * ± 0.79	3.68 ^a^ ± 0.83

* Comparison between before the pandemic and during the pandemic showed statistical differences for all variables (worse during the pandemic period); Different lowercase letters inside each column and for each variable show statistically different results (*p* < 0.05); y/o—years old.; MW—Minimum Wage in Brazil (June 7th 2020)—U$ 213.0.

## Supplementary Materials

The following are available online at https://www.mdpi.com/1660-4601/17/15/5541/s1, Table S1. Wellbeing at work by factors and by socioeconomic and demographic variables of Brazilian dietitians before and during the SARS-COV-2 pandemic period (*n* = 1359).

## Appendix A. Brazilian-Portuguese Questionnaire to Evaluate the Dietitians’ Wellbeing at Work before and during the SARS-COV-2 Pandemic

### Appendix A.1. Questionário de Avaliação Do Bem-Estar Do Nutricionista No Trabalho Antes E Durante A Pandemia da Sars-Cov-2/Nutritionist’s Wellbeing Assessment Questionnaire at Work before and during the Sars-Cov-2 Pandemic

A seção seguinte destina-se a recolher dados dos participantes da investigação, com a finalidade de permitir a análise de tendências de respostas em função de características pessoais e do trabalho. Por favor complete o questionário com estes dados, com a garantia de que nenhuma destas informações será utilizada para identificar qualquer participante da investigação./*The next section is intended to collect data from the research participants, in order to allow the analysis of trends in responses according to personal and work characteristics. Please complete the questionnaire with this data, ensuring that none of this information will be used to identify any research participants.*

1. Sexo Biológico/*Gender*
2. Idade/*Age*
3. Estado civil/*Marital status*
4. Qual o estado brasileiro de residência atual?/*What is your resindency state in Brazil?*
5. Qual a sua religião?/*Religion*
6. Quantas pessoas vivem na sua casa incluindo você? *Counting with you, how many persons live in the house*?
7. Qual a sua renda familiar?/*Family income*
8. Você tem filhos?/*Do you have children*?
9. Qual o seu nível educacional?/*Educational level*
10. Qual a sua área de atuação? (você pode marcar mais de uma opção)/*What is your area of working as a dietitian?*
11. Em quantos locais você exerce atividade como nutricionista?/*In how many places do you work as a dietitian?*
12. Há quanto tempo terminou sua graduação em nutrição?/*How long ago did you graduate in nutrition?*
13. Em que tipo de instituição cursou sua graduação em nutrição?/*In what type of university did you study nutrition?*
14. Você continua trabalhando no período da pandemia da SARS-CoV-2?/*Do you comtinue working during SARS-CoV-2 pandemic?*
15. Você testou positivo para a SARS-CoV-2?/*Did you test positive for SARS-CoV-2?*
16. Alguma pessoa da sua família testou positivo para a SARS-CoV-2?/*Has anyone in your family tested positive for SARS-CoV-2*

### Appendix A.2. Escala de Bemestar Individual No Trabalho/Wellbeing at Work

Este instrumento pretende avaliar o seu nível de bem-estar no exercício da profissão de nutricionista. Para tanto, você deve avaliar cada uma das afirmativas abaixo, preenchendo os espaços em branco conforme os códigos seguintes:

Nunca/*never* (0); Raramente/*Rarely* (1); Às Vezes/*Sometimes* (2); Frequentemente/*Frequently* (3); Sempre/*Always* (4)

1. O trabalho como nutricionista é importante para mim/*My work as a dietitian is important for me*
2. Percebo que minha profissão é valorizada onde trabalho/*I realize that my profession is valued where I work*
3. Considero que exerço um trabalho importante para a sociedade*/I comsider my work important for society*
4. Sou recompensado(a) por minha competência como nutricionista/*I am rewarded for my competence as a dietitian*
5. Sou admirado(a) por meus colegas pelo trabalho que faço/*I am admired by my colleagues for my work*
6. Tenho liberdade para executar minhas atividades com meu estilo pessoal/*I am free to carry out my activities in my personal style*
7. Tenho a infraestrutura material necessária para a execução do meu trabalho/*I have the necessary material infrastructure to carry out my work*
8. Tenho a possibilidade de me desenvolver profissionalmente*/I have the possibility to improve professionally*
9. Sinto-me realizado(a) profissionalmente/*I feel professionally fulfilled*
10. Sinto-me seguro(a) com a possibilidade de continuar trabalhando como nutricionista/*I feel safe with the possibility of continuing to work as a dietitian*
11. Tenho um bom suporte tecnológico para executar o meu trabalho/*I have good technological support to do my job*
12. As relações sociais com meus colegas influenciam positivamente o meu trabalho/*Social relations with my colleagues positively influence my work*
13. Sinto-me bem com o relacionamento com meus chefes/*I feel good about my relationship with my bosses*
14. Sinto-me bem com o relacionamento com meus subordinados/*I feel good about my relationship with my employees*
15. Considero justo o salário que recebo/*My salary is fare*
16. Tenho orgulho de pertencer à categoria profissional de nutricionista/*I am proud to be part of the dietitans professional category.*
17. Sinto-me bem trabalhando como nutricionista./*I feel good to be working as a dietitian*
18. Sou admirado pela sociedade/clientes pelo trabalho que faço*/I am admired by society/clients for the work I peform*
19. Considero meu trabalho criativo e estimulante/*I consider my work criative and challenging*
20. Quero permanecer sempre trabalhando como nutricionista/*I want to continue working as a dietitian*
21. Considero que as tarefas que executo para atender meus clientes são importantes para a qualidade de vida deles/*I believe that the tasks I perform to my clients are important to their quality of life*
22. Sinto-me estimulado(a) a estar sempre atualizado(a)/*I feel encouraged to always be up to date*
23. Considero que os avanços da ciência da nutrição melhoram o meu desempenho profissional*/I believe that advances in nutrition science improve my professional performance*
24. Considero que as novas tecnologias criadas melhoram meu desempenho profissional*/I believe that the new technologies improve my professional performance*
25. Considero minha carga horária de trabalho adequada/*I consider my workload adequate*

## References

[1] Cao W., Fang Z., Hou G., Han M., Xu X., Dong J., Zheng J. (2020). The psychological impact of the COVID-19 epidemic on college students in China. Psychiatry Res..

[2] (2020). World Health Organization Coronavirus disease (COVID-19)—Situation Report—139.

[3] (2020). Worldometer Coronavirus Cases. Worldometer.

[4] Jeyabaladevan P. (2020). COVID-19: An FY1 on the frontline. Med. Educ. Online.

[5] Satici B., Saricali M., Satici S.A., Griffiths M.D. (2020). Intolerance of Uncertainty and Mental Wellbeing: Serial Mediation by Rumination and Fear of COVID-19. Int. J. Ment. Health Addict..

[6] APA Professional Practice Guidelines for Integrating the Role of Work and Career into Psychological Practice. https://www.apa.org/practice/guidelines/role-work-career.

[7] Blustein D.L. (2008). The Role of Work in Psychological Health and Wellbeing: A Conceptual, Historical, and Public Policy Perspective. Am. Psychol..

[8] Ibrahim N.M., Khogali N.A., Mahmoud H., Fatahi H. (2019). Job satisfaction of dietitians in government hospitals Khartoum State. Int. J. Home Sci..

[9] Gingras J., De Jonge L.A., Purdy N. (2010). Prevalence of dietitian burnout. J. Hum. Nutr. Diet..

[10] Real H., Bento A., Graça P., De Nutricionistas A.P. (2011). A Profissão do nutRicionista em Portugal: Evolução e Regulamentação Profissional.

[11] Real H., Craveiro C. (2014). Past, Present and Future Perspectives on the Profession of Nutritionist in Portugal. Rev. Nutr..

[12] De Akutsu R.C. (2008). Brazilian dieticians: Professional and demographic profiles. Rev. Nutr..

[13] Ferreira M.C., De Castro Coelho L., Asakura L., Molina Cohrs F., Sachs A., Sávio K., De Cássia Akutsu R. (2014). Relationship Between Social and Personal Variables, Body Image, and Wellbeing at Work of Nutritionists. Rev. Colomb. Psicol..

[14] Brasil (2020). LEI No 14.023, DE 8 DE JULHO DE 2020.

[15] Paz M.G.T., Gosendo E.E.M., Dessen M.C., Mourão R.G.V. (2009). Justiça Organizacional e Bem-Estar Pessoal nas Organizações. Rev. EVS—Rev. Ciênc. Ambient. Saúde.

[16] Akutsu R.D.C., Da Paz G.T., De Almeida C.C. (2011). Valores y bienestar de los dietistas brasileños Values and wellbeing of brazilian dieticians. Rev. Latinoam. Psicol..

[17] WHO (2010). Healthy Workplace Framework and Model: Background and Supporting Literature and Practice.

[18] Chen Q., Liang M., Li Y., Guo J., Fei D., Wang L., He L., Sheng C., Cai Y., Li X. (2020). Mental health care for medical staff in China during the COVID-19 outbreak. Lancet Psychiatry.

[19] Yang Y., Li W., Zhang Q., Zhang L., Cheung T., Xiang Y.T. (2020). Mental health services for older adults in China during the COVID-19 outbreak. Lancet Psychiatry.

[20] CFN Conselho Federal de Nutricionistas—Estatísticas. https://www.cfn.org.br/index.php/estatistica/.

[21] Hair J.F., Black W.C., Babin B.J., Anderson R.E., Tatham R.L. (2009). Análise Multivariada de Dados.

[22] Stacey G. (2020). The place of person-centred care in an international response to the Covid-19 pandemic. Int. Pract. Dev. J..

[23] Mo Y., Deng L., Zhang L., Lang Q., Liao C., Wang N., Qin M., Huang H. (2020). Work stress among Chinese nurses to support Wuhan in fighting against COVID-19 epidemic. J. Nurs. Manag..

[24] IBGE Desemprego. https://www.ibge.gov.br/explica/desemprego.php.

[25] Coetzee S.K., Klopper H.C. (2010). Compassion fatigue within nursing practice: A concept analysis. Nurs. Health Sci..

[26] Mcdonald G., Jackson D., Vickers M.H., Wilkes L. (2016). Surviving workplace adversity: A qualitative study of nurses and midwives and their strategies to increase personal resilience. J. Nurs. Manag..

[27] Najjar N., Davis L.W., Beck-Coon K., Carney Doebbeling C. (2009). Compassion fatigue: A review of the research to date and relevance to cancer-care providers. J. Health Psychol..

[28] Cheung R.B., Aiken L.H., Clarke S.P., Sloane D.M. (2008). Nursing care and patient outcomes: International evidence. Enferm. Clin..

[29] Brazil Governo está Cadastrando Profissionais para o Enfrentamento da Covid-19—Português (Brasil). https://www.gov.br/pt-br/noticias/saude-e-vigilancia-sanitaria/2020/04/governo-esta-cadastrando-profissionais-para-o-enfrentamento-da-covid-19.

[30] Brazil 500 mil Profissionais Dispostos a Atuarem no Combate ao Coronavírus. https://www.saude.gov.br/noticias/agencia-saude/46805-500-mil-profissionais-dispostos-a-atuarem-no-combate-ao-coronavirus.

[31] CFN Conselho Federal de Nutricionistas. https://www.cfn.org.br/.

[32] Real H., Ávila H. (2012). Thirty Years of the Portuguese Association of Nutritionists: An Historical Profile and Memories. Nutrícias.

[33] Campos F.M., Kraemer F.B., Machado P.A.N., Carvalho M.C.V.S., Prado S.D. (2016). Gender and profession: Considerations on female roles in building the nutritionist career. Demetra Food. Nutr. Health.

[34] Pless A.M., Wolman P.G., Stallings S.F., Goodner C.H. (1998). Job Satisfaction of South Carolina Dietitians. J. Am. Diet. Assoc..

[35] Meyer R., Gilroy R., Williams P. (2002). Dietitians in New South Wales: Workforce trends 1984–2000. Aust. Health Rev..

[36] Conselho Federal de Nutricionistas (2019). Inserção Profissional dos Nutricionistas no Brasil.

[37] Sauer K., Canter D., Shanklin C. (2010). Job Satisfaction of Dietitians with Management Responsibilities: An Exploratory Study Supporting ADA’s Research Priorities. J. Am. Diet. Assoc..

[38] Chin J.H., You J.S., Chang K.J. (2012). Comparison of Role Conflict, Self-Efficacy, Job Satisfaction, and Job Involvement between Nutrition Teachers and Dietitians at School Food Service in Incheon Metropolitan City—Focusing on the Interactions between Nutrition Teachers and Dietitians. Korean J. Nutr..

[39] Gaião A.F.C. (2013). A Satisfação no Trabalho Percecionada pelo Dietista/Nutricionista.

[40] Brasil Coronavírus Brasil. https://covid.saude.gov.br/.

[41] Brazil (2020). MEDIDA PROVISÓRIA No. 936, DE 1o DE ABRIL DE 2020.

[42] Brazil (2020). MEDIDA PROVISÓRIA No. 927, DE 22 DE MARÇO DE 2020.

[43] VanderWeele T. (2017). On the Promotion of Human Flourishing. Proc. Natl. Acad. Sci. USA.

[44] Wilcox W.B. (2002). A Report from Family Scholars T HIS STATEMENT Comes from a Team of Family Scholars Chaired by.

[45] McKee-Ryan F.M., Song Z., Wanberg C.R., Kinicki A.J. (2005). Psychological and physical wellbeing during unemployment: A meta-analytic study. J. Appl. Psychol..

[46] Ryff C.D., Heidrich S.M. (1997). Experience and wellbeing: Explorations on domains of life and how they matter. Int. J. Behav. Dev..

[47] Carneiro P., Heckman J., Vytlacil E. (2010). Estimating Marginal Returns to Education. NBER Working Paper No. 16474. Natl. Bur. Econ. Res..

[48] Zuckerman P. (2006). Atheism: Contemporary numbers and patterns. The Cambridge Companion to Atheism.

[49] King S.D.W., Fitchett G., Berry D.L. (2013). Screening for religious/spiritual struggle in blood and marrow transplant patients. Support. Care Cancer.

[50] Sedikides C. (2010). Why does religiosity persist?. Personal. Soc. Psychol. Rev..

[51] Kortt M.A., Dollery B., Grant B. (2015). Religion and Life Satisfaction Down Under. J. Happiness Stud..

[52] Ferreira A.V., Pinto M.D.C., Neto F. (2012). Religiosidade E Bem-Estar em Estudantes Portugueses, Moçambicanos, Angolanos e Brasileiros.

[53] IBGE Instituto Brasileiro de Geografia e Estatística Produto Interno Bruto—PIB. https://www.ibge.gov.br/explica/pib.php.

