# Supplementary material for: Wellbeing at Work before and during the SARS-COV-2 Pandemic: A Brazilian Nationwide Study among Dietitians
Source: Int J Environ Res Public Health. 2020 Jul 31;17(15):5541. doi: 10.3390/ijerph17155541 (PMC7432096; doi:10.3390/ijerph17155541)
Supplement: Supplementary file 1 [file ijerph-17-05541-s001.pdf]

Table S1. Well-being at work by factors and by socioeconomic and demographic variables of Brazilian dietitians before and during the pandemic period (n = 1359).

**VARIABLE**		**Before pandemic Mean±SD**				**During pandemic Mean±SD**			
		Factor 1	Factor 2	Factor 3	Factor 4	Factor 1	Factor 2	Factor 3	Factor 4
Gender	Female	3.46±0.87	4.08±0.68	4.08±0.78	3.91±0.93	3.25±0.94	3.93±0.77	3.98±0.85	3.68±1.01
	Male	3.50±0.90	4.11±0.69	4.06±0.79	3.90±0.90	3.28±0.95	3.96±0.82	3.91±0.95	3.56±1.09
Age group	21 to 24 y/o	3.41±0.90	4.05±0.75	4.06±0.79	3.82±0.99	3.14±0.95	3.87±0.85	3.90±0.92	3.56±1.12
	25 to 29 y/o	3.44±0.86	4.09±0.61	4.10±0.73	3.96±0.85	3.24±0.90	3.93±0.71	4.00±0.82	3.68±0.98
	30 to 34 y/o	3.51±0.83	4.11±0.68	4.13±0.75	3.93±0.92	3.30±0.92	3.97±0.80	4.01±0.82	3.68±1.02
	35 to 39 y/o	3.48±0.83	4.14±0.62	4.12±0.74	3.96±0.89	3.28±0.92	3.99±0.77	4.00±0.86	3.71±0.98
	40 to 44 y/o	3.45±0.96	3.96±0.79	3.94±0.92	3.88±1.00	3.27±0.98	3.84±0.80	3.89±0.93	3.72±1.00
	45 to 49 y/o	3.39±0.91	4.03±0.75	4.02±0.87	3.82±1.02	3.15±1.00	3.87±0.79	3.89±0.91	3.53±1.14
	50 to older	3.47±0.92	4.11±0.67	4.08±0.80	3.89±0.95	3.30±0.96	3.97±0.71	4.01±0.84	3.69±1.00
Brazilian region	North	3.43±0.92	4.11±0.64	4.13±0.73	3.91±0.90	3.23±0.97	3.95±0.75	4.02±0.83	3.66±1.02
	Northeast	3.52±0.87	4.13±0.66	4.11±0.78	3.94±0.92	3.27±0.96	3.96±0.76	3.97±0.86	3.67±1.00
	Midwest	3.48±0.89	4.10±0.72	4.11±0.81	3.99±0.92	3.30±0.94	3.97±0.80	4.02±0.87	3.7±1.04
	Southeast	3.4±0.85	4.01±0.66	4.02±0.76	3.79±0.96	3.21±0.90	3.87±0.74	3.94±0.82	3.59±1.04
	South	3.41±0.83	4.04±0.72	4.00±0.82	3.87±0.92	3.22±0.89	3.87±0.79	3.88±0.92	3.62±0.99
Level of education (highest degree)	Undergraduate	3.51±0.89	4.15±0.64	4.14±0.78	3.98±0.91	3.31±0.94	3.99±0.77	4.02±0.89	3.70±1.04
	Graduate/Residency	3.46±0.88	4.00±0.70	4.07±0.79	3.90±0.91	3.24±0.94	3.91±0.77	3.95±0.87	3.67±0.99
	Master´s	3.41±0.87	4.06±0.68	4.06±0.81	3.86±0.99	3.21±0.92	3.90±0.77	3.97±0.83	3.60±1.07
	PhD	3.35±0.88	3.92±0.73	4.00±0.76	3.74±0.97	3.29±0.95	3.96±0.75	4.02±0.77	3.73±1.02
Marital status	Without partner	3.46±0.85	4.07±0.67	4.09±0.78	3.90±0.89	3.25±0.91	3.91±0.74	3.9±0.86	3.66±1.02
	With partner	3.46±0.89	4.09±0.69	4.08±0.78	3.89±0.95	3.26±0.95	3.95±0.78	3.98±0.86	3.68±1.02
Children	Yes	3.46±0.88	4.07±0.69	4.04±0.79	3.87±0.94	3.27±0.93	3.93±0.76	3.96±0.86	3.68±0.99
	No	3.46±0.87	4.10±0.68	4.11±0.78	3.94±0.92	3.24±0.94	3.93±0.78	3.99±0.86	3.66±1.04
Family monthly income	≤ 1 MW	3.43±0.88	3.93±0.80	4.10±0.65	4.00±0.80	3.3±0.96	3.89±0.78	3.97±0.76	3.85±0.80
	> 1 to 2 MW	3.55±0.93	4.19±0.65	4.24±0.79	4.08±0.86	3.3±1.00	4.04±0.79	4.12±0.86	3.83±0.95
	>2 to 3 MW	3.49±0.86	4.11±0.66	4.07±0.78	3.92±0.90	3.27±0.88	3.95±0.79	3.96±0.86	3.69±0.99
	>3 to 5 MW	3.44±0.88	4.04±0.73	4.05±0.84	3.87±0.94	3.22±0.95	3.88±0.79	3.93±0.92	3.59±1.06
	> 5 to 10 MW	3.41±0.85	4.06±0.64	4.04±0.80	3.88±0.96	3.24±0.89	3.93±0.71	3.96±0.85	3.67±1.01

	> 10 to 20 MW	3.50±0.90	4.14±0.68	4.14±0.70	3.94±0.91	3.27±1.01	3.95±0.80	4.00±0.81	3.66±1.05
	> 20 MW	3.45±0.93	4.07±0.76	4.06±0.76	3.84±0.93	3.26±1.00	3.92±0.84	3.99±0.86	3.60±1.06
Area of Practice	Clinic	3.40±0.91	4.06±0.69	4.08±0.77	3.89±0.93	3.16±0.95	3.88±0.76	3.95±0.86	3.61±1.04
	Teaching	3.47±0.92	4.08±0.73	4.1±0.67	3.93±0.91	3.34±1.00	3.98±0.78	4.03±0.74	3.73±1.00
	Foodservice administration	3.48±0.91	4.09±0.70	4.1±0.82	3.93±0.92	3.31±0.97	4±0.78	4.05±0.86	3.76±1.01
	Public health	3.27±0.94	3.96±0.72	3.91±0.87	3.73±1.02	3.04±1.00	3.76±0.81	3.73±0.95	3.42±1.13
	More than one area of practice	3.52±0.83	4.11±0.65	4.09±0.77	3.93±0.92	3.3±0.89	3.95±0.76	3.98±0.85	3.68±1.00
	Others	3.53±0.79	4.17±0.68	4.2±0.83	4.11±0.82	3.35±0.85	4.09±0.72	4.16±0.84	3.94±0.83
Number of workplaces	1	3.44±0.89	4.08±0.71	4.06±0.81	3.91±0.95	3.25±0.95	3.95±0.79	3.97±0.85	3.69±1.03
	2	3.45±0.84	4.09±0.62	4.14±0.70	3.90±0.90	3.22±0.90	3.9±0.73	3.98±0.85	3.61±1.03
	3	3.63±0.82	4.15±0.64	4.11±0.81	3.95±0.86	3.34±0.93	3.92±0.75	3.94±0.93	3.64±0.98
	> 3	3.54±0.86	4.10±0.65	4.07±0.85	3.99±0.81	3.4±0.89	3.99±0.79	4.01±0.90	3.79±0.98
Type of institution	Private	3.47±0.86	4.08±0.70	4.06±0.78	3.90±0.91	3.27±0.91	3.92±079	3.94±0.87	3.65±1.01
	Public	3.45±0.89	4.09±0.67	4.11±0.78	3.93±0.94	3.24±0.96	3.94±0.75	4.01±0.84	3.69±1.02
Time undergraduate completion	≤ 2 years	3.46±0.93	4.09±0.71	4.09±0.80	3.90±0.96	3.24±0.97	3.91±0.82	3.96±0.90	3.61±1.10
	>2 to 5 years	3.46±0.88	4.07±0.71	4.08±0.80	3.93±0.89	3.25±0.93	3.94±0.76	4.00±0.82	3.68±0.96
	>5 to 10 years	3.46±0.80	4.06±0.66	4.08±0.79	3.88±0.91	3.21±0.88	3.87±0.78	3.93±0.87	3.61±1.01
	>10 to 15 years	3.50±0.84	4.14±0.64	4.16±0.68	3.97±0.87	3.36±0.89	4.04±0.73	4.06±0.78	3.79±0.95
	>15 years	3.43±0.91	4.07±0.68	4.02±0.83	3.89±0.97	3.23±0.98	3.92±0.74	3.93±0.90	3.67±1.04
Do you continue working during COVID-19?	no	3.47±0.86	4.07±0.73	4.03±0.83	3.84±0.93	3.24±0.92	3.91±0.80	3.92±0.91	3.61±1.00
	yes in person	3.41±0.93	4.07±0.70	4.08±0.80	3.90±0.95	3.24±0.98	3±0.77	3.96±0.86	3.64±1.03
	yes in person with some adaptations	3.49±0.85	4.10±0.65	4.10±0.77	3.94±0.93	3.26±0.92	3.92±0.76	3.97±0.88	3.69±1.03
	yes remotely	3.46±0.87	4.09±0.67	4.09±0.76	3.93±0.91	3.27±0.93	3.96±0.76	4.01±0.82	3.70±1.01
Did you test positive for COVID-19?	No	3.46±0.87	4.08±0.76	4.08±0.78	3.91±0.92	3.25±0.93	3.94±0.77	3.97±0.86	3.67±1.01
	Yes	3.54±0.92	4.13±0.78	4.09±0.77	3.88±0.97	3.31±1.00	3.89±0.85	3.96±0.88	3.55±1.11
Did any family members test positive for COVID-19?	No	3.46±0.87	4.09±0.80	4.09±0.78	3.92±0.93	3.26±0.93	3.94±0.77	3.98±0.86	3.67±1.03
	Yes (does not live with me)	3.46±0.87	4.12±0.82	4.08±0.69	3.95±0.91	3.27±0.91	3.99±0.61	4.02±0.71	3.76±1.00

Yes (living with me)	3.43±0.94	4.03±0.88	4.04±0.82	3.87±0.88	3.21±0.99	3.89±0.80	3.91±0.90	3.64±0.98
